# Urothelial carcinoma of the fossa navicularis successfully treated with laser ablation and distal urethrectomy: A case report

**DOI:** 10.5339/qmj.2021.60

**Published:** 2021-10-25

**Authors:** Khalid Assadiq, Ahmad Rimawi, Khaled Jebreen

**Affiliations:** ^1^Consultant Urologist: The Specialty Hospital, Amman, Jordan E-mail: ahmad_rimawi2010@hotmail.com; ^2^Medical Student: University of Jordan, Faculty of Medicine, Amman 11942, Jordan; ^3^Urology specialist, specialty hospital, Amman, Jordan

**Keywords:** transitional cell carcinoma, laser ablation, penis neoplasms

## Abstract

Primary urothelial carcinomas very rarely arise from the fossa navicularis of the penis. They are rarely reported in the literature, with only 13 cases reported thus far. Herein, we present the case of a 34-year-old man with bloody urethral discharge due to a mass detected by cystourethroscopy in the fossa navicularis. Biopsy confirmed the diagnosis of noninvasive urothelial carcinoma. The patient was managed successfully with two sessions of holmium laser ablation, followed by distal urethrectomy. After the treatment, the patient's erectile function and continence were preserved, and no tumor recurrence was observed after 1 year of follow-up.

## Introduction

Primary urethral cancers are rare and account for less than 1% of all urogenital cancers.^1^ Urothelial carcinomas extremely rarely arise from the fossa navicularis of the penis, and to the best of our knowledge, only 13 cases of such tumors were reported in the English literature.^2^ Urethral stricture, radiation therapy, and urethroplasty were described as risk factors for such tumors.^3^ Retrospective studies have also revealed an association of urothelial carcinoma of the bladder with the *Human papillomavirus*. However, such an association could not be thoroughly evaluated in urothelial carcinomas arising from the fossa navicularis because of their rarity.^3,4^ Most cases of urothelial carcinomas of the fossa navicularis were treated with nonconservative methods that led to a significant loss of erectile function.

Herein, we report a case of primary urothelial carcinoma of the fossa navicularis that presented with bloody urethral discharge. It was managed with laser ablation and distal urethrectomy that fully preserved the erectile function.

## Case Presentation

A 34-year-old man presented to our outpatient clinic with a chief complaint of bloody discharge and painful erection and ejaculation. He also reported occasional greenish discharge from the urethra. At 6 months before presentation, he had undergone cystoscopy, which was reported as “free.” Physical examination results of the external genitalia were normal. Urine analysis showed pyuria with no hematuria consistent with a urinary tract infection. He was given a course of levofloxacin, and cystourethroscopy was scheduled.

**Figure 1.**
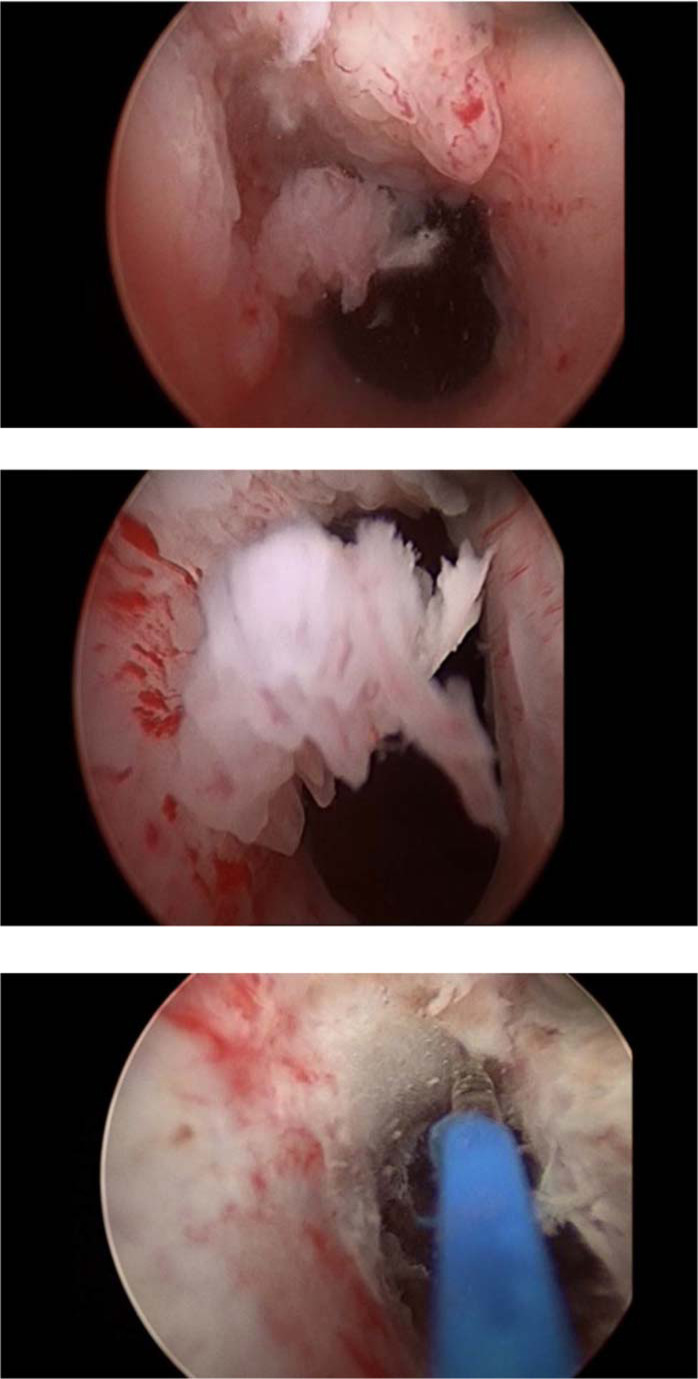
Cystoscopy showing a large papillary mass in the fossa navicularis.

Several months before presentation, the patient underwent multiple cystoscopies with negative results, and no tumors were detected. At 2 months later, cystourethroscopy was performed in an erection state to mimic his symptoms, which mainly occurred with erections. To induce an erection before cystoscopy, the patient was injected with prostaglandin-E1 locally before the procedure. Upon advancement of the cystoscope, we fund a large papillary mass measuring 5 mm ×  6 mm within the fossa navicularis (Figure 1). With further advancement of the cystoscope, we found normal bladder mucosa with no evidence of any masses or transitional cell carcinoma elsewhere. Multiple biopsies were taken from the tumor, tumor base, prostate, and bladder. The tumor in the fossa navicularis was also ablated using a low-power holmium laser.

**Figure 2.**
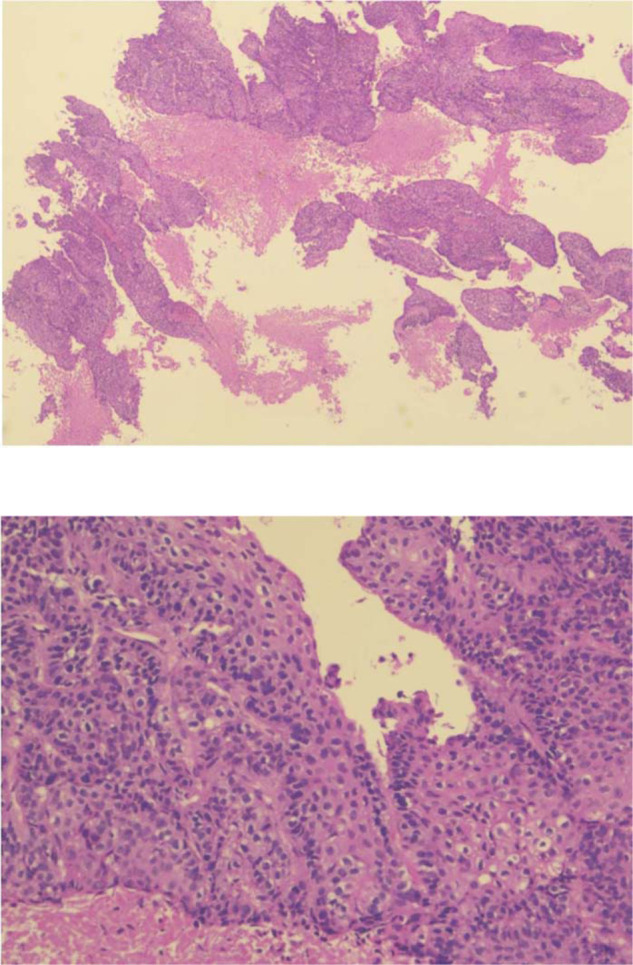
Biopsy with hematoxylin and eosin stain showing high-grade noninvasive urothelial carcinoma.

After the procedure, the biopsied tissue was sent for analysis, and histopathological examination showed the tumor as noninvasive high-grade papillary urothelial carcinoma (Figure 2). Computed tomography urography did not find masses or signs of malignancy elsewhere in the urinary system.

Cystourethroscopy was repeated after 3 weeks along with an excisional biopsy of the superficial inguinal lymph nodes. Biopsy was taken from the base of the tumor in the fossa navicularis, and laser cautery ablation was repeated with a high-power holmium laser setting. Histopathological examination revealed high-grade urothelial carcinoma with squamous differentiation and metastasis to the right superficial inguinal lymph nodes.

The patient underwent a metastatic workup with positron-emission tomography (PET), which showed no evidence of metastasis to the chest, abdomen, or pelvis. Distal urethrectomy with penile urethrostomy was performed after PET, and the resected tissue was sent for a frozen section procedure. Histopathological examination showed no signs of urothelial carcinoma, and the whole tissue specimen was free from tumor. The patient was referred to the oncology department for further management. He received three cycles of cisplatin-based chemotherapy and two PET scans over 1 year, which showed no signs of recurrence. The treatment preserved both the erectile function and continence of the patient.

## Discussion

Primary urethral carcinomas are rare but aggressive tumors that account for less than 1% of all genitourinary malignancies.^1^ Transitional cell carcinomas account for only 15% of male urethral carcinomas and mostly arise from the prostatic and membranous urethra. By contrast, urethral cancers that arise in the bulbar and spongy urethra are squamous cell carcinomas because the distal urethral segments are only lined by squamous epithelium.^5^ Urothelial carcinomas arising from the fossa navicularis are extremely rare because this structure does not normally contain urothelial epithelium; thus, such a tumor unusually arises from this area.^6^ Two hypotheses were proposed to explain the etiology of such tumors. First, the tumor might have originated from an ectopic focus of urothelial epithelium in the fossa navicularis, and second, a metaplastic urothelial transformation in the fossa navicularis may be the precursor for such tumors.^7^

In the literature, bloody urethral discharge was the most common presentation of urothelial carcinoma of the fossa navicularis. Multiple treatment options were reported in the literature, with transurethral resection as the most common, followed by partial or total penectomy.^2^ However, the presented case is one of the few cases in which such a tumor was managed with laser ablation, followed by distal urethrectomy. This approach allowed complete excision of the tumor with total preservation of erectile function, and this was very important given the young age of our patient. Moreover, we did not expect a tumor-free specimen during distal urethrectomy. The present case indicated that ablation with the holmium laser alone was sufficient for the complete removal of the tumor.

## Conclusions

This report highlights a case of urothelial carcinoma that was managed with laser excision and distal urethrectomy, which completely removed the tumor and preserved the erectile function of the patient.
